# Antimalarial activity of *Garcinia mangostana* L rind and its synergistic effect with artemisinin in vitro

**DOI:** 10.1186/s12906-017-1649-8

**Published:** 2017-02-28

**Authors:** Susy Tjahjani

**Affiliations:** Faculty of Medicine, Maranatha Christian University, Bandung, Indonesia

**Keywords:** *Garcinia mangostana*, Artemisinin, Antimalarial, Synergism, in vitro

## Abstract

**Background:**

Malaria especially falciparum malaria still causes high morbidity and mortality in tropical countries. Several factors have been linked to this situation and the most important one is the rapid spread of parasite resistance to the currently available antimalarials, including artemisinin. Artemisinin is the main component of the currently recommended antimalarial, artemisinin based combination therapy (ACT), and it is a free radical generating antimalarial. *Garcinia mangostana* L (mangosteen) rind contain a lot of xanthone compounds acting as an antioxidant and exhibited antimalarial activity. The aim of this study was to evaluate the antimalarial activity of mangosteen rind extract and its fractions and their interaction with artemisinin against the 3D7 clone of *Plasmodium falciparum* in vitro.

**Methods:**

Dry ripe mangosteen rind was extracted with ethanol followed by fractionation with hexane, ethylacetate, buthanol, and water consecutively to get ethanol extract, hexane, athylacetate, buthanol, and water fractions. Each of these substances was diluted in DMSO and examined for antimalarial activity either singly or in combination with artemisinin in vitro against *Plasmodium falciparum* 3D7 clone. Synergism between these substances with artemisinin was evaluated according to certain formula to get the sum of fractional inhibitory concentration 50 (∑FIC_50_).

**Results:**

Analysis of the parasite growth in vitro indicated that IC_50_ of these mangosteen rind extract, hexane, ethylacetate, buthanol, and water fraction ranged from 0.41 to > 100 μg/mL. All of the ∑FIC50 were <1.

**Conclusions:**

This study demonstrated a promising antimalarial activity of the extract and fractions of *G.mangostana* L rind and its synergistic effect with artemisinin. Further study using lead compound(s) isolated from extract and fractions should be performed to identify more accurately their mechanism of antimalarial activities.

**Electronic supplementary material:**

The online version of this article (doi:10.1186/s12906-017-1649-8) contains supplementary material, which is available to authorized users.

## Background

Malaria remains a major public health issue worldwide despite a decreasing trend in its morbidity and mortality between 2000 and 2015. In 2015, WHO reported 214 million clinical cases with 438.000 death and approximately 3.3 billion population or half of the world population are at risk. Most malaria cases were caused by *Plasmodium falciparum* and *Plasmodium vivax* but most of the death was caused by *Plasmodium falciparum* infection [1]. The persistently high morbidity and mortality of malaria is due to the rapid speed of drug resistant parasite including the currently used artemisinin combination therapy (ACT) [2].

Artemisinin, the main component of ACT, is a free radical generating antimalarial [3] that has a short half life [4–6], and rapidly clear the parasite [7]. Its single prescription is not recommended due to recrudescence rate [8], and therefore several partner drugs with longer half life are now available such as in artemeter-lumefantrine, dihydroartemisinin-piperaquine, artesunate-mefloquine, artesunate-amodiaquine. Unfortunately resistance of the parasite to the partner drugs has also been reported [9–11].

Xanthones are potent antioxidant [12], and they possibly reduce the free radical over production in malaria especially if artemisinin is used to manage the disease. On the other side, these compounds can also inhibit heme polymerization [13] that is needed by the parasite to detoxify the heme over production. Our previous study revealed that alpha-mangostin and gamma-mangostin are both xanthone compounds, and exhibited antimalarial activities with synergistic effect with artemisinin [14].

*Garcinia mangostana* L (mangosteen) grows in tropical area [15], where malaria is endemic. Its general name is mangosteen (English), manggis (Indonesia), and its taxonomic profile is: Magnoliophyta division, Magnoliopsida class, Dilleniidae subclass, Theales order, Clusiaceae family, Garcinia genus, *Garcinia mangostana* L. species. Its rind, usually a waste product, contained a lot of xanthone compounds [16, 17] and therefore may be developed as alternative drug to treat malaria. This study aims to explore the potential of mangosteen rind as partner drug of artemisinin for treating malaria.

## Methods

### Plant collection and preparation

Identification of this plant was done by Mr. Djuandi, a curator at the Herbarium Bandungense, Sekolah Tinggi Imu Hayati, Bandung Institute of Technology (ITB), Bandung, Indonesia. A voucher specimen of this material has been deposited in a publicly available herbarium, the Herbarium Bogoriense, Research Center of Biology, Indonesian Institute of Sciences by Dr. J S Rahajoe in 2012 with deposition number of 1143/IPH.1.02/lf.8/VII/2012. The fresh ripe *G.mangostana* L fruit which had purple color was collected from Subang District, West Java, Indonesia. The fruit was washed with tap water gently and its rind without kernel and seed inside was carefully analyzed for debris and content. The rind was cut into small pieces, air dried, and pulverized into powder. The powder was then macerated with absolute ethanol and subsequently evaporated to obtain the paste like extract according to standard procedure [18]. The extract was then fractionated using hexane to obtain hexane fraction following the same procedure [18]. The hexane fraction obtained was then re-fractionated using ethylacetate to obtain ethylacetate fraction. This procedure was continued using buthanol and water consecutively to obtain buthanol and water fraction. All of these extract and fractions were stored in the - 20 °C freezer until used. To examine the antimalarial activity, each of these substances was dissolved in dimethyl sulfoxide (DMSO, Sigma Aldrich, IL, USA) to make a stock solution separately.

### Parasite cultivation and determination of 50% Inhibitory Concentration (IC_50_) of *G. mangostana* L rind extracts, hexane, ethylacetate, buthanol, and water fractions against *P. falciparum* 3D7 clone

To determine the antimalarial activity of these extracts and fractions, malarial parasites, *P. falciparum* 3D7 clone was obtained from the Malaria Laboratory, The Eijkman Institute for Molecular Biology, Jakarta, and was propagated in vitro in duplicate in a 24 well culture plate in the presence of a wide concentration ranges of each extracts and fractions following the procedure described previously [19]. The Red Blood Cell (RBC) used for the propagation of the parasites was a left over or outdated RBC provided by the Indonesian Red Cross, Surabaya, Indonesia without any personal identity except for the type of blood. The parasites concentration in vitro was calculated before and after 48 h incubation with a wide concentration range of each of the extracts and fractions by determining the amount of parasites per 5000 RBC in Giemsa stained thin blood smear. The parasites growth inhibition was calculated by comparing the parasites concentrations of the treated group with the untreated control. The parasites IC_50_ of each of the extracts and fractions was determined using probit analysis. The antimalarial activity was classified following the criteria as describe previously [20].

### Determination of interaction between artemisinin and *G. mangostana* L rind extracts, hexane, ethylacetate, buthanol, and water fractions as antimalarial against *P. falciparum* 3D7 clone in vitro

The parasites was cultivated in duplicate in the presence of a wide concentration range of combination of artemisinin and each of these extract sand fractions in 1:1 concentration ratio. The parasites growth before and after 48 h incubation was evaluated by measurement of the parasites concentration in Giemsa stained thin blood smear and the growth inhibition as well as the IC_50_ was determined using the aforementioned procedure. Interaction between artemisinin and each of these extracts and fractions was determined according to the sum of fractional 50% inhibitory concentration (FIC_50_) of artemisinin and each of these extracts and fractions according to formula: A_c_/A_s_ + B_c_/B_s_, where A_c_ and B_c_ are the concentration of A and B in the combination associated with a particular level of effect, e.g., IC_50_, while A_s_ and B_s_ are the concentration of A and B when are used singly to produced the same level of effect. If this sum is 1, the interaction of these drugs is named additive interaction. Synergistic interaction is named if this sum is less than 1 and if this sum is more than 1, it is named antagonistic interaction [21].

## Results

### Proximate analysis

Proximate analysis of dry *G.mangostana* L rind is shown in Additional file 1. The rind extracts mainly contained carbohydrate, crude fibre, and ash.

### In vitro antimalarial activity of *G. mangostana* L extracts and its fractions against 3D7 clone of *P. falciparum*

The parasites growth in the presence of different concentration of its extracts, hexane, ethylacetate, buthanolic, and water fractions is shown in Additional files 2, 3, 4, 5 and 6 respectively. Analysis of the parasites growth revealed that the IC_50_ of the extracts, hexane, ethylacetate, buthanolic, and water fractions, ranged from 0.42 μg/mL (ethanolic extract), 0.12 μg/mL (hexane fraction), 1–10 μg/mL (ethylacetate fraction), 1152 μg/mL (buthanolic fraction) to > 100 μg/mL (water fraction).

### In vitro interaction between artemisinin and *G. mangostana* L rind extract and its fractions as antimalaria against 3D7 clone of *P. falciparum*

The parasite growth in the presence of combination of artemisinin and the extract, hexane, ethylacetate, buthanolic, and water fraction is shown in (Additional files 7, 8, 9, 10 and 11). Analysis of the parasite growth revealed the IC_50_ ranged from 0,00001 to 0,0001 μg/mL.

## Discussion

### In vitro antimalarial activity of *G.mangostana* L rind extract and its fractions

The present study demonstrates a promising antimalarial activity of the extract, hexane, and ethylacetate fraction of the rind of *G.mangostana* L with the IC_50_ of less than 10 μg/mL. However, the buthanolic and water fractions revealed a very weak antimalarial activities. The results of this study therefore deserves further exploration to identify the lead compounds that may underline the antimalarial activity. The *G.mangostana* L. rind contains many kinds of phenolic compounds such as tannins, anthocyanins, xanthones, and their derivates [22–25]. The most abundant xanthones in *G.mangostana* L. rind are alpha-mangostin and gamma-mangostin [26]. These xanthones and other xanthones such as garcinone C and garcinone D also existed in the rind, and have been reported to exhibit active antimalarial activities [14]. Therefore we may conclude that the antimalarial activity exhibited by the rind extract and fractions are caused by the existence of alpha-mangostin, gamma-mangostin, garcinone C, and garcinone D in the rind. Further, the antimalarial activity of xanthones was previously associated with the interference with the heme polymerizationin the malarial parasite [13]. It was reported that xanthones form soluble complex with heme dimmers so that it increases osmotic pressure in the parasite food vacuole causing parasite lysis and death [27]. *G. mangostana* L rind ethanolic extract also interrupts the tricarboxylic acid (TCA) metabolism of the parasite as indicated by the absence of malate product in the culture medium [28].

### In vitro interaction between artemisinin and *G. mangostana* L rind extract and its fractions as antimalaria against 3D7 clone of *P. falciparum*

All kinds of combination of the extract and fractions with artemisinin showed a very strong antimalarial activity as indicated by the IC_50_ which was < 0,001 μg/mL and the sum of FIC_50_ which was in the range of 0,03 – 0,25, which means synergistic interaction (Additional file 12). Similar finding was also reported in our previous in vitro study using pure compounds of alpha-mangostin, gamma-mangostin, garcinone C and garcinone D [14]. Other in vitro study also demonstrated that the synergistic effect between hydroxycalabaxanthone and artesunate [29]. As the studies using the relatively pure compounds, we therefore could suggest that the synergistic antimalarial activity exhibited in our study using extract and fractions are caused by the existence of similar compounds.

## Conclusion

This study demonstrated a promising antimalarial activity and its synergistic antimalarial activity of the extract and fractions of *G.mangostana* L rind with artemisinin. Further study using lead compound(s) isolated from extract and fractions should be performed to identify more accurately their mechanism of antimalarial activities.

## Additional files

Additional file 1: Table S1. Proximate analysis of *G. mangostana* L rind. (DOC 29 kb)
Additional file 2: Table S2. Parasite growth and inhibition rate in *G.mangostana* L rind extract treatment in vitro. (DOC 41 kb)
Additional file 3: Table S3. Parasite growth and inhibition rate in *G.mangostana* L rind hexane fraction treatment in vitro*.* (DOC 41 kb)
Additional file 4: Table S4. Parasite growth and inhibition rate in *G.mangostana* L rind ethylacetate fraction treatment in vitro. (DOC 40 kb)
Additional file 5: Table S5. Parasite growth and inhibition rate in *G.mangostana* L rind buthanol fraction treatment in vitro. (DOC 41 kb)
Additional file 6: Table S6. Parasite growth and inhibition rate in *G.mangostana* L rind water fraction treatment in vitro. (DOC 40 kb)
Additional file 7: Table S7. Parasite growth and inhibition rate in *G.mangostana* L rind extract + artemisinin treatment in vitro. (DOC 42 kb)
Additional file 8: Table S8. Parasite growth and inhibition rate in *G.mangostana* L rind hexane fraction + artemisinin treatment in vitro. (DOC 42 kb)
Additional file 9: Table S9. Parasite growth and inhibition rate in *G.mangostana* L rind ethylacetate fraction + artemisinin treatment in vitro. (DOC 42 kb)
Additional file 10: Table S10. Parasite growth and inhibition rate in *G.mangostana* L rind buthanolic fraction+ artemisinin treatment in vitro. (DOC 42 kb)
Additional file 11: Table S11. Parasite growth and inhibition rate in *G.mangostana* L rind water fraction + artemisinin treatment in vitro. (DOC 42 kb)
Additional file 12: Table S12. Interaction between artemisinin and *G. mangostana* L rind extract and its fractions as antimalaria against 3D7 clone of *P. Falciparum* in vitro [30]. (DOC 30 kb)

## Abbreviations

ACT: Artemisinin based combination therapy
DMSO: Dimethylsulfoxide
FIC_50_: Fractional 50% inhibitory concentration
IC_50_: 50% inhibitory concentration
TCA: Tricarboxylic acid cycle
