# Supplementary material for: Antimalarial activity of Garcinia mangostana L rind and its synergistic effect with artemisinin in vitro
Source: BMC Complement Altern Med. 2017 Feb 28;17:131. doi: 10.1186/s12906-017-1649-8 (PMC5329916; doi:10.1186/s12906-017-1649-8)
Supplement: Additional file 1: Table S1. — Proximate analysis of G. mangostana L rind. (DOC 29 kb) [file 12906_2017_1649_MOESM1_ESM.doc]

**Additional file 1**

**Table S1 Proximate analysis of *G. mangostana*** L rind

| No | Component | Percentage |
| --- | --- | --- |
| 1 | Water | 10.31 |
| 2 | Ash | 20.54 |
| 3 | Protein | 3.43 |
| 4 | Crude fiber | 25.53 |
| 5 | Crude fat | 0.54 |
| 6 | Carbohydrate | 49.96 |
