# Supplementary material for: Antimalarial activity of Garcinia mangostana L rind and its synergistic effect with artemisinin in vitro
Source: BMC Complement Altern Med. 2017 Feb 28;17:131. doi: 10.1186/s12906-017-1649-8 (PMC5329916; doi:10.1186/s12906-017-1649-8)
Supplement: Additional file 2: Table S2. — Parasite growth and inhibition rate in G.mangostana L rind extract treatment in vitro. (DOC 41 kb) [file 12906_2017_1649_MOESM2_ESM.doc]

**Additional file 2**

**Table S2 Parasite growth and inhibition rate in *G.mangostana* L rind extract treatment *in vitro***

| extract  (µg/mL) | Parasitemia (%) | | parasite growth rate (%) | Parasite growth inhibition rate (%) | Average of parasite growth inhibition rate (%) | IC_50_  (µg/mL) |
| --- | --- | --- | --- | --- | --- | --- |
|  | 0 hour | 48 hours |  |  |  |  |
| Negative control | 1.04 | 6.63 | 5.59 | - | - | 0.415 |
|  | 1.04 | 6.45 | 5.41 | - |  |  |
| 100 | 1.04 | 0 | 0 | 100 | 100 |  |
|  | 1.04 | 0 | 0 | 100 |  |  |
| 10 | 1.04 | 1.20 | 0.16 | 97.14 | 98.57 |  |
|  | 1.04 | 0.93 | 0 | 100 |  |  |
| 1 | 1.04 | 3.93 | 2.89 | 48.30 | 50.68 |  |
|  | 1.04 | 3.58 | 2.54 | 53.05 |  |  |
| 0.1 | 1.04 | 4.88 | 3.84 | 31.31 | 33.40 |  |
|  | 1.04 | 4.53 | 3.49 | 35.49 |  |  |
| 0.01 | 1.04 | 6.35 | 5.31 | 5.09 | 4.21 |  |
|  | 1.04 | 6.27 | 5.23 | 3.32 |  |  |
