# Supplementary material for: Antimalarial activity of Garcinia mangostana L rind and its synergistic effect with artemisinin in vitro
Source: BMC Complement Altern Med. 2017 Feb 28;17:131. doi: 10.1186/s12906-017-1649-8 (PMC5329916; doi:10.1186/s12906-017-1649-8)
Supplement: Additional file 3: Table S3. — Parasite growth and inhibition rate in G.mangostana L rind hexane fraction treatment in vitro. (DOC 41 kb) [file 12906_2017_1649_MOESM3_ESM.doc]

**Additional file 3**

**Table S3 Parasite growth and inhibition rate in *G.mangostana* L rind hexane fraction treatment *in vitro***

| Hexane fraction  (µg/mL) | Parasitemia (%) | | parasite growth rate (%) | Parasite growth inhibition rate (%) | Average of parasite growth inhibition rate (%) | IC50  (µg/mL) |
| --- | --- | --- | --- | --- | --- | --- |
|  | 0 hour | 48 hours |  |  |  |  |
| Negative control | 1.04 | 5.44 | 4.4 | - | - | 0.12 |
|  | 1.04 | 5.42 | 4.38 | - |  |  |
| 100 | 1.04 | 1.48 | 0.44 | 90 | 94.32 |  |
|  | 1.04 | 1.10 | 0.06 | 98.63 |  |  |
| 10 | 1.04 | 1.99 | 0.95 | 78.41 | 80.53 |  |
|  | 1.04 | 1.80 | 0,76 | 82.65 |  |  |
| 1 | 1.04 | 2.47 | 1.43 | 67.5 | 72.34 |  |
|  | 1.04 | 2.04 | 1 | 77.17 |  |  |
| 0.1 | 1.04 | 3.48 | 2.44 | 44.54 | 47.16 |  |
|  | 1.04 | 3.24 | 2.20 | 49.77 |  |  |
| 0.01 | 1.04 | 4.42 | 3.38 | 23.18 | 28.72 |  |
|  | 1.04 | 3.92 | 2.88 | 34.25 |  |  |
