# Supplementary material for: Antimalarial activity of Garcinia mangostana L rind and its synergistic effect with artemisinin in vitro
Source: BMC Complement Altern Med. 2017 Feb 28;17:131. doi: 10.1186/s12906-017-1649-8 (PMC5329916; doi:10.1186/s12906-017-1649-8)
Supplement: Additional file 4: Table S4. — Parasite growth and inhibition rate in G.mangostana L rind ethylacetate fraction treatment in vitro. (DOC 40 kb) [file 12906_2017_1649_MOESM4_ESM.doc]

**Additional file 4**

**Table S4 Parasite growth and inhibition rate in *G.mangostana* L rind ethylacetate fraction treatment *in vitro***

| Ethylacetate fraction  (µg/mL) | Parasitemia (%) | | parasite growth rate (%) | Parasite growth inhibition rate (%) | Average of parasite growth inhibition rate (%) | IC50  (µg/mL) |
| --- | --- | --- | --- | --- | --- | --- |
|  | 0 hour | 48 hours |  |  |  |  |
| Negative control | 1.04 | 6.55 | 5.51 | - | - | 1-10 |
|  | 1.04 | 6.26 | 5.22 | - |  |  |
| 100 | 1.04 | 0.09 | 0 | 100 | 100 |  |
|  | 1.04 | 0.09 | 0 | 100 |  |  |
| 10 | 1.04 | 1.22 | 0.18 | 96.55 | 98.28 |  |
|  | 1.04 | 1.02 | 0 | 100 |  |  |
| 1 | 1.04 | 5.90 | 4.86 | 11.80 | 16.06 |  |
|  | 1.04 | 5.20 | 4.16 | 20.31 |  |  |
| 0.1 | 1.04 | 7.79 | 6.75 | 0 | 0 |  |
|  | 1.04 | 7.16 | 6.12 | 0 |  |  |
| 0.01 | 1.04 | 6.58 | 5.54 | 0 | 0 |  |
|  | 1.04 | 6.40 | 5.36 | 0 |  |  |
