# Supplementary material for: Antimalarial activity of Garcinia mangostana L rind and its synergistic effect with artemisinin in vitro
Source: BMC Complement Altern Med. 2017 Feb 28;17:131. doi: 10.1186/s12906-017-1649-8 (PMC5329916; doi:10.1186/s12906-017-1649-8)
Supplement: Additional file 5: Table S5. — Parasite growth and inhibition rate in G.mangostana L rind buthanol fraction treatment in vitro. (DOC 41 kb) [file 12906_2017_1649_MOESM5_ESM.doc]

**Additional file 5**

**Table S5 Parasite growth and inhibition rate in *G.mangostana* L rind buthanol fraction treatment *in vitro***

| Buthanol fraction  (µg/mL) | Parasitemia (%) | | parasite growth rate (%) | Parasite growth inhibition rate (%) | Average of parasite growth inhibition rate (%) | IC50  (µg/mL) |
| --- | --- | --- | --- | --- | --- | --- |
|  | 0 hour | 48 hours |  |  |  |  |
| Negative control | 1.04 | 6.18 | 5.14 | - | - | 1153.028 |
|  | 1.04 | 6.47 | 5.43 | - |  |  |
| 100 | 1.04 | 4.39 | 3.35 | 34.82 | 35.55 |  |
|  | 1.04 | 4.50 | 3.46 | 36.28 |  |  |
| 10 | 1.04 | 4.67 | 3.63 | 29.38 | 30.53 |  |
|  | 1.04 | 4.75 | 3.71 | 31.68 |  |  |
| 1 | 1.04 | 4.87 | 3.83 | 29.47 | 28.73 |  |
|  | 1.04 | 4.95 | 3.91 | 27.99 |  |  |
| 0.1 | 1.04 | 5.10 | 4.06 | 21.01 | 22.20 |  |
|  | 1.04 | 5.20 | 4.16 | 23.39 |  |  |
| 0.01 | 1.04 | 5.94 | 4.90 | 4.67 | 4.54 |  |
|  | 1.04 | 6.23 | 5.19 | 4.42 |  |  |
