# Supplementary material for: Antimalarial activity of Garcinia mangostana L rind and its synergistic effect with artemisinin in vitro
Source: BMC Complement Altern Med. 2017 Feb 28;17:131. doi: 10.1186/s12906-017-1649-8 (PMC5329916; doi:10.1186/s12906-017-1649-8)
Supplement: Additional file 6: Table S6. — Parasite growth and inhibition rate in G.mangostana L rind water fraction treatment in vitro. (DOC 40 kb) [file 12906_2017_1649_MOESM6_ESM.doc]

**Additional file 6**

**Table S6 Parasite growth and inhibition rate in *G.mangostana* L rind water fraction treatment *in vitro***

| Water fraction  (µg/mL) | Parasitemia (%) | | parasite growth rate (%) | Parasite growth inhibition rate (%) | Average of parasite growth inhibition rate (%) | IC50  (µg/mL) |
| --- | --- | --- | --- | --- | --- | --- |
|  | 0 hour | 48 hours |  |  |  |  |
| Negative control | 1.04 | 6.14 | 5.10 | - | - | >100  (no activity) |
|  | 1.04 | 6.06 | 5.02 | - |  |  |
| 100 | 1.04 | 4.01 | 2.97 | 41.76 | 44.38 |  |
|  | 1.04 | 3.70 | 2.66 | 47.01 |  |  |
| 10 | 1.04 | 4.74 | 3.70 | 27.45 | 27.57 |  |
|  | 1.04 | 4.67 | 3.63 | 27.69 |  |  |
| 1 | 1.04 | 7.24 | 6.20 | 0 | 0 |  |
|  | 1.04 | 7.14 | 6.10 | 0 |  |  |
| 0.1 | 1.04 | 6.80 | 5.76 | 0 | 0 |  |
|  | 1.04 | 7.11 | 6.07 | 0 |  |  |
| 0.01 | 1.04 | 7.15 | 6.11 | 0 | 0 |  |
|  | 1.04 | 7.20 | 6.16 | 0 |  |  |
