# Supplementary material for: Antimalarial activity of Garcinia mangostana L rind and its synergistic effect with artemisinin in vitro
Source: BMC Complement Altern Med. 2017 Feb 28;17:131. doi: 10.1186/s12906-017-1649-8 (PMC5329916; doi:10.1186/s12906-017-1649-8)
Supplement: Additional file 7: Table S7. — Parasite growth and inhibition rate in G.mangostana L rind extract + artemisinin treatment in vitro. (DOC 42 kb) [file 12906_2017_1649_MOESM7_ESM.doc]

**Additional file 7**

**Table S7 Parasite growth and inhibition rate in *G.mangostana* L rind extract + artemisinin treatment *in vitro***

| Ext + art  (µg/mL) | Parasitemia (%) | | Parasite growth rate (%) | Parasite growth inhibition rate (%) | Average of parasite growth inhibition rate (%) | IC_50_  (µg/mL) |
| --- | --- | --- | --- | --- | --- | --- |
|  | 0 hour | 48 hours |  |  |  |  |
| Negative control | 0.91 | 4.78 | 3.87 | - |  | 0.001 – 0.0001 |
|  | 0.91 | 4.51 | 3.60 | - |  |  |
| 0.1 | 0.91 | 0 | 0 | 100 | 100 |  |
|  | 0.91 | 0 | 0 | 100 |  |  |
| 0.01 | 0.91 | 0 | 0 | 100 | 100 |  |
|  | 0.91 | 0 | 0 | 100 |  |  |
| 0.001 | 0.91 | 0 | 0 | 100 | 100 |  |
|  | 0.91 | 0 | 0 | 100 |  |  |
| 0.0001 | 0.91 | 6.49 | 5.58 | 0 | 0 |  |
|  | 0.91 | 5.21 | 4.30 | 0 |  |  |
| 0.00001 | 0.91 | 6.40 | 5.49 | 0 | 0 |  |
|  | 0.91 | 4.87 | 3.96 | 0 |  |  |

Notes: ext + art =*G.mangostana* L rind extract + artemisinin
