# Supplementary material for: Antimalarial activity of Garcinia mangostana L rind and its synergistic effect with artemisinin in vitro
Source: BMC Complement Altern Med. 2017 Feb 28;17:131. doi: 10.1186/s12906-017-1649-8 (PMC5329916; doi:10.1186/s12906-017-1649-8)
Supplement: Additional file 8: Table S8. — Parasite growth and inhibition rate in G.mangostana L rind hexane fraction + artemisinin treatment in vitro. (DOC 42 kb) [file 12906_2017_1649_MOESM8_ESM.doc]

**Additional file 8**

**Table S8 Parasite growth and inhibition rate in *G.mangostana* L rind hexane fraction + artemisinin treatment *in vitro***

| Hex + art  (µg/mL) | Parasitemia (%) | | Parasite growth rate (%) | Parasite growth inhibition rate (%) | Average of parasite growth inhibition rate (%) | IC_50_  (µg/mL) |
| --- | --- | --- | --- | --- | --- | --- |
|  | 0 hour | 48 hours |  |  |  |  |
| Negative control | 0.91 | 6.00 | 5,09 | - |  | 0.0001 – 0.00001 |
|  | 0.91 | 5.50 | 4,59 | - |  |  |
| 0.1 | 0.91 | 0 | 0 | 100 | 100 |  |
|  | 0.91 | 0 | 0 | 100 |  |  |
| 0.01 | 0.91 | 0 | 0 | 100 | 100 |  |
|  | 0.91 | 0 | 0 | 100 |  |  |
| 0.001 | 0.91 | 0.04 | 0 | 100 | 100 |  |
|  | 0.91 | 0.03 | 0 | 100 |  |  |
| 0.0001 | 0.91 | 3.47 | 2,56 | 49.70 | 53,28 |  |
|  | 0.91 | 2.89 | 1,98 | 56.86 |  |  |
| 0.00001 | 0.91 | 4.24 | 3.33 | 34.58 | 31.67 |  |
|  | 0.91 | 4.18 | 3.27 | 28.76 |  |  |

Notes: hex + art = *G.mangostana* L rind hexane fraction + artemisinin
