# Supplementary material for: Antimalarial activity of Garcinia mangostana L rind and its synergistic effect with artemisinin in vitro
Source: BMC Complement Altern Med. 2017 Feb 28;17:131. doi: 10.1186/s12906-017-1649-8 (PMC5329916; doi:10.1186/s12906-017-1649-8)
Supplement: Additional file 9: Table S9. — Parasite growth and inhibition rate in G.mangostana L rind ethylacetate fraction + artemisinin treatment in vitro. (DOC 42 kb) [file 12906_2017_1649_MOESM9_ESM.doc]

**Additional file 9**

**Table S9 Parasite growth and inhibition rate in *G.mangostana* L rind ethylacetate fraction + artemisinin treatment *in vitro***

| EA + art  (µg/mL) | Parasitemia (%) | | Parasite growth rate (%) | Parasite growth inhibition rate (%) | Average of parasite growth inhibition rate (%) | IC_50_  (µg/mL) |
| --- | --- | --- | --- | --- | --- | --- |
|  | 0 hour | 48 hours |  |  |  |  |
| Negative control | 0.91 | 5.44 | 4.53 | - | - | 0.001 – 0.0001 |
|  | 0.91 | 5.20 | 4.29 | - |  |  |
| 0.1 | 0.91 | 0 | 0 | 100 | 100 |  |
|  | 0.91 | 0 | 0 | 100 |  |  |
| 0.01 | 0.91 | 0 | 0 | 100 | 100 |  |
|  | 0.91 | 0 | 0 | 100 |  |  |
| 0.001 | 0.91 | 0 | 0 | 100 | 100 |  |
|  | 0.91 | 0 | 0 | 100 |  |  |
| 0.0001 | 0.91 | 3.38 | 2.47 | 45.47 | 47.21 |  |
|  | 0.91 | 3.10 | 2.19 | 48.95 |  |  |
| 0.00001 | 0.91 | 4.40 | 3.49 | 22.96 | 21.97 |  |
|  | 0.91 | 4.30 | 3.39 | 20.98 |  |  |

Notes: EA + art = *G.mangostana* L rind ethylacetate fraction+ artemisinin
