# Supplementary material for: Antimalarial activity of Garcinia mangostana L rind and its synergistic effect with artemisinin in vitro
Source: BMC Complement Altern Med. 2017 Feb 28;17:131. doi: 10.1186/s12906-017-1649-8 (PMC5329916; doi:10.1186/s12906-017-1649-8)
Supplement: Additional file 10: Table S10. — Parasite growth and inhibition rate in G.mangostana L rind buthanolic fraction+ artemisinin treatment in vitro. (DOC 42 kb) [file 12906_2017_1649_MOESM10_ESM.doc]

**Additional file 10**

**Table S10 Parasite growth and inhibition rate in *G.mangostana* L rind buthanolic fraction+ artemisinin treatment *in vitro***

| Buth + art  (µg/mL) | Parasitemia (%) | | Parasite growth rate (%) | Parasite growth inhibition rate (%) | Average of parasite growth inhibition rate (%) | IC_50_  (µg/mL) |
| --- | --- | --- | --- | --- | --- | --- |
|  | 0 hour | 48 hours |  |  |  |  |
| Negative control | 0.91 | 4.51 | 3.60 | - | - | 0.001 – 0.0001 |
|  | 0.91 | 4.04 | 3.13 | - |  |  |
| 0.1 | 0.91 | 0 | 0 | 100 | 100 |  |
|  | 0.91 | 0 | 0 | 100 |  |  |
| 0.01 | 0.91 | 0 | 0 | 100 | 100 |  |
|  | 0.91 | 0 | 0 | 100 |  |  |
| 0.001 | 0.91 | 0.05 | 0 | 100 | 100 |  |
|  | 0.91 | 0.06 | 0 | 100 |  |  |
| 0.0001 | 0.91 | 4.68 | 3.77 | 0 | 0 |  |
|  | 0.91 | 4.53 | 3.62 | 0 |  |  |
| 0.00001 | 0.91 | 4.73 | 3.82 | 0 | 0 |  |
|  | 0.91 | 4.51 | 3.60 | 0 |  |  |

Notes: buth + art = *G.mangostana* L rind buthanolic fraction+ artemisinin
