# Supplementary material for: Antimalarial activity of Garcinia mangostana L rind and its synergistic effect with artemisinin in vitro
Source: BMC Complement Altern Med. 2017 Feb 28;17:131. doi: 10.1186/s12906-017-1649-8 (PMC5329916; doi:10.1186/s12906-017-1649-8)
Supplement: Additional file 11: Table S11. — Parasite growth and inhibition rate in G.mangostana L rind water fraction + artemisinin treatment in vitro. (DOC 42 kb) [file 12906_2017_1649_MOESM11_ESM.doc]

**Additional file 11**

**Table S11 Parasite growth and inhibition rate in *G.mangostana* L rind water fraction + artemisinin treatment *in vitro***

| water + art  (µg/mL) | R | % Parasitemia | | Parasite growth rate (%) | Parasite growth inhibition rate (%) | Average of parasite growth inhibition rate (%) | IC_50_  (µg/mL) |
| --- | --- | --- | --- | --- | --- | --- | --- |
|  |  | 0 hour | 48 hours |  |  |  |  |
| Negative control | 1 | 0.91 | 5.40 | 4.49 | - | - | 0.001 – 0.0001 |
|  | 2 | 0.91 | 5.01 | 4.10 | - |  |  |
| 0.1 | 1 | 0.91 | 0 | 0 | 100 | 100 |  |
|  | 2 | 0.91 | 0 | 0 | 100 |  |  |
| 0.01 | 1 | 0.91 | 0 | 0 | 100 | 100 |  |
|  | 2 | 0.91 | 0 | 0 | 100 |  |  |
| 0.001 | 1 | 0.91 | 0 | 0 | 100 | 100 |  |
|  | 2 | 0.91 | 0 | 0 | 100 |  |  |
| 0.0001 | 1 | 0.91 | 3.12 | 2.21 | 50.78 | 49.05 |  |
|  | 2 | 0.91 | 3.07 | 2.16 | 47.32 |  |  |
| 0.00001 | 1 | 0.91 | 3.55 | 2.64 | 41.20 | 41.09 |  |
|  | 2 | 0.91 | 3.33 | 2.42 | 40.98 |  |  |

Notes: water + art = *G.mangostana* L rind water fraction + artemisinin
