# Supplementary material for: Antimalarial activity of Garcinia mangostana L rind and its synergistic effect with artemisinin in vitro
Source: BMC Complement Altern Med. 2017 Feb 28;17:131. doi: 10.1186/s12906-017-1649-8 (PMC5329916; doi:10.1186/s12906-017-1649-8)
Supplement: Additional file 12: Table S12. — Interaction between artemisinin and G. mangostana L rind extract and its fractions as antimalaria against 3D7 clone of P. Falciparum in vitro [30]. (DOC 30 kb) [file 12906_2017_1649_MOESM12_ESM.doc]

**Additional file 12**

**Table S12 Interaction between artemisinin and *G. mangostana* L rind extract and its fractions as antimalaria against 3D7 clone of *P. Falciparum in vitro***

| TYPE OF COMBINATION | IC_50_ (µg/ mL) | ∑FIC_50_ | INTERACTION |
| --- | --- | --- | --- |
| *G.mangostana* L rind extract + artemisinin | 0.001 -0.0001 | 0.251 | synergistic |
| Hexane Fraction + artemisinin | 0.0001 -0.00001 | 0.026 | synergistic |
| Ethyl acetate Fraction + artemisinin | 0.001 – 0.0001 | 0.250 | synergistic |
| Buthanolic Fraction + artemisinin | 0.001 – 0.0001 | 0.250 | synergistic |
| Water Fraction + artemisinin | 0.001 – 0.0001 | 0.250 | Synergistic |

Notes: IC_50_ of artemisinin against 3D7 clone of Plasmodium falciparum = 0.0096 µg/ mL [30]
